# Risk Factors Linked to Depression After Treatment in Cancer Survivors in Jeddah, Saudi Arabia

**DOI:** 10.7759/cureus.12710

**Published:** 2021-01-14

**Authors:** Adel Hajjay, Shoroq Abduljabbar M Hassan, Rida Rayes, Lujain Alzahrani, Khalid F Alotaibi, Shurooq Alharbi

**Affiliations:** 1 Medicine and Surgery, Batterjee Medical College, Jeddah, SAU

**Keywords:** cancer survivorship, post-treatment, symptoms, social economic status, depression, quality of life, fear of recurrence, risk factors

## Abstract

Background

While many cancer patients survive long after diagnosis, one is bound to experience long-term and latent side effects as a result of the treatment. This experience results in a poor quality of life, morbidity, and mortality for cancer survivors. Understanding how to manage these side-effects is quite important and a key to cancer survivorship.

Objective

Given the importance of this area, the main objective of this study seeks to understand the risk factor associated with cancer survivorship.

Methods

A cross-sectional study was conducted among 154 cancer survivors from King Fahad General and Kind Abdul-Aziz hospitals. Descriptive and inferential statistics were used in analyzing the responses. Descriptive statistics were used in understanding the extent of the risk factors while inferential statistics were used to understand the cross-relationships.

Results

This study included 154 cancer survivors aged 18 and above at King Fahad Hospital and King Abdul-Aziz Hospital in Jeddah, Makkah Region, KSA. Thirty-one (20.1%) of the sample were males and 123 (79.9%) were females. Of all the survivors who screened, 111 (72.9%) symptoms occur before treatment, 116 (75.3%) within the treatment, and 57 (37.09%) after treatment. The relationship between Socioeconomic Status Cross Symptoms categories demonstrated below includes accommodation type, education level, age, gender, and job.

Conclusion

The study concludes that post-cancer treatment symptoms are highly affected by other factors such as social-economic status as well as family support. As a result, the study recommends the establishment of educational materials on post-treatment symptoms, care-plans, and support groups for cancer survivors.

## Introduction

Depression in cancer survivors is a psychiatric disorder and common comorbidity affecting >20% of survivors [1]. It affects how the person feels, thinks, and acts. Actually, it affects all aspects of a person's life and leads to many complications that if left untreated may result in a poor quality of life and thoughts of death or suicide [2].

Several factors may contribute to depression including age and gender. Bevilacqua et al. did a study in 2017 that included over 1012 survivors of different types of cancer who were referred to the Survivorship Clinics at Memorial Sloan Kettering Cancer Center to find out if there is a relation between age and gender to depression among cancer survivors. They found there are no differences in depression between older (65 years old and above) and younger adult (30-55 years old) cancer survivors but there is a higher risk for the younger aged group (identify the age group) to develop depression. While in the case of gender, women reported greater rates of depression score than men [3]. While another study was done by Champion et al. in 2015 which compared younger (diagnosed at age 45 years or before) and older breast cancer survivors (diagnosed between 55 and 70 years) and age-matched controls on the specific and overall quality of life domains. The study revealed that younger survivors reported more depression than age-matched controls (p<0.001) and reported more depressive symptoms (p<0.001) than older survivors [4]. Another study done by Miaskowski did a study on 50-year-old and older cancer patients from both genders who are receiving active treatment for their illness. The study evaluated the gender and the age of the patients as a risk factor for the severity of depression among them. They used the Center for Epidemiologic Studies Depression (CES-D) scale and they found as men increased in age one year their depression decreased by 0.33 units while for women, as they increase in age one year the depression increased by 0.16 units [5]. Also, the educational level may have a relation to depression among cancer survivors [6]. Further studies observed patients who graduate from the university had higher life levels than others. There is a poor quality of life when the education level was low [7] because a high level of education will bring a good job with higher salaries and more intellectual and social activities which helps in improving quality of life after treatment [8]. An Italian study of 405 breast cancer survivors with a median time since the treatment of eight years found that women with higher education had a slightly higher physical function compared to women with shorter education. As a result of higher physical function, quality of life improves later on and decrease the chance of anxiety and depression [6]. Previous research on the employment status of cancer survivors has indicated that cancer may not affect survivors' job and they are usually able to return to work, but there are a group of cancer survivors who experience impairment in health as a result of their illness, and this impairment sometimes leads to a decrease in their ability to work or even to a disability, which may lead to depression [9]. Furthermore, accumulating data over the past decade indicate a significant relationship between socioeconomic status and depression in cancer survivors. Data show a high association between low socioeconomic status and depression which may be due to less access to medical resources [10]. Also, a previous study has demonstrated the social support of cancer patients negatively related to depression, as well as predicting the mental health of the patient [11]. In addition to social support, a depressed mood in the spouse of a cancer survivor can increase the risk of development of depression in the cancer survivor. Litzelman and Yabroff did a study between 2004 and 2012 on these spouses and their relation to the development of depression and they found, the cancer survivor whose spouse reported a depressed mood was 4.27 times more to develop depression, especially female survivor. While those who reported a better mood were 30% less depressed [12]. Another factor is the fear of the recurrence of cancer. New literature shows the fear of cancer recurrence (FCR) is a problem among survivors. FCR is known as a long-term problem among survivors as it continues to be a reminder of their traumatic experiences associated with diagnosis and treatment. According to The American Cancer Society, at least 70% of survivors experience FCR [13]. Moreover, different types of cancer may be a factor causing depression in cancer survivors. According to the World Health Organization "country profile of Saudi Arabia 2014," breast cancer is the most common cancer type among females in Saudi Arabia [14]. A study done in 2,130 patients with breast cancer who have undergone a mastectomy in Korea by using data from the Korean Health Insurance Review and Assessment Service (HIRA), shows that patients undergoing mastectomy for breast cancer experience depression more than healthy people. However, young adults overcome their depressive mood symptoms more quickly than middle-aged and older adults [15]. Mastectomy-induced-body image impairment generates higher rates of depression [16]. In colorectal cancer, which is the most common cancer of males in Saudi Arabia [14], the prevalence of depression in survivors appears to be related closely to the physical functioning, financial concerns, cognitive functioning, fear of recurrence, and lack of social support. Moreover, being an older age male was associated with more depression [17,18].

Different modalities of treatment may pose another risk factor in causing depression in cancer survivors. Depression in chemotherapy-treated cancer patients has received increased attention in psycho-oncology research during the past decade [19]. Some medications that are used in the treatment of chemotherapy-associated nausea-like haloperidol have been implicated in causing depression by reducing dopaminergic transmission in the brain, and therefore, development of depressive symptoms [20]. As was identified previously, mastectomy can induce depression in breast cancer survivors. Many studies were done on women and their adaptation after different surgical procedures and one of them was a study done in 1957 on breast cancer survivors one to five years after diagnosis. The method used is a self-report questionnaire that assesses many factors including quality of life, body image, sexual, and physical functioning. The study divided the women into three groups: women who underwent a lumpectomy, women who underwent mastectomy alone, and women who had a mastectomy with reconstruction. The results show women who underwent mastectomy and reconstruction had a negative influence on their sex lives (45.4% in comparison with 29.8% for lumpectomy and 41.3% for mastectomy alone; P=0.0001) [21]. As there is a lack of studies addressing the relationship between various risk factors linked to depression in cancer survivors, this study aims to identify these factors and their relation to depression in cancer survivors in Jeddah, KSA.

## Materials and methods

Research methodology

Research Design

Set to determine the risk factors associated with cancer treatment, a cross-sectional study was undertaken. The study was exploratory in nature and mostly used descriptive statistics in determining the spread and prevalence of cancer-related risk factors.

Participants, Sample Size, and Selection

The study used a sample of 154 cancer survivors at King Fahad Hospital and Abdul-Aziz hospital in Jeddah, Makkah Region, KSA.

Data Collection Methods

Data collection was done through self-administered questionnaires and the cancer survivors were interviewed by a research group.

Data Analysis Plan

The analysis was done by testing the significant relationship between the symptoms that cancer survivors were experiencing and the economic, social, and educational status that they were exposed to. In addition, the analysis determined if there is a relationship between the type of cancer the cohort had and their thinking in negative ways such as thinking about suicide, obsessive-compulsive disorder, and fear of recurrence.

The study also looked into the relationship between family, friends, social media, and health providers' support that cancer survivors received during their journey as well as their psychological state, their negative thoughts, and type of treatment they had. Statistical analysis was done using the Chi-square test of nominal data. Simple descriptive statistics are reported as proportions for qualitative variables such as frequencies and percentages of applied treatment types on cancer survivors and their psychological state. The results were considered significant with P<0.05.

## Results

Data analysis

Findings

Out of the 154 participants, 20.1% were male while 79.9% were female. The participants were between 18 and 30 years old, 30 and 40 years old, and 40 and 50 years old, each accounted for 24.7%. While 50-60 years old, 60-70 years old, and above 70 years old accounting for 13.6%, 10.4%, and 1.9%, respectively. Over 88.3% of the participants were Saudi nationals while 11.7% were of other nationalities. Most of the participants had completed the University level of education and accounted for 54.2% (Table 1).

**Table 1:** Demographic data description SAR: Saudi Riyal.

Demographic data	N %
Gender	
Male	31 (20.1%)
Female	123 (79.9%)
Age	
18–30 years	38 (24.7%)
30–40 years	38 (24.7%)
40–50 years	38 (24.7%)
50–60 years	21 (13.6%)
60–70 years	16 (10.4%)
Above than 70 years	3 (1.9%)
Education level	
Elementary school	11 (7.2%)
Secondary school	9 (5.9%)
High school	41 (26.8%)
University	83 (54.2%)
High education	9 (5.9%)
Nationality	
Saudi	136 (88.3%)
Non-Saudi	18 (11.7%)
Job	
Governmental job	40 (26.1%)
Private sector employee	7 (4.6%)
Freelancer	4 (2.6%)
Retired	22 (14.4%)
Student	27 (17.6%)
Unemployed	53 (34.6%)
Accommodation type	
Rented house	49 (32.0%)
Owned house	104 (68.0%)
House type	
Apartment	79 (51.6%)
Villa	68 (44.4%)
Shelter	4 (2.6%)
Palace	2 (1.3%)
Income	
Less than 3,000 SAR	35 (23.8%)
3,000–5,000 SAR	33 (22.4%)
5,000–10,000 SAR	34 (23.1%)
10,000–15,000 SAR	20 (13.6%)
15,000–20,000 SAR	18 (12.2%)
Above 20,000 SAR	7 (4.8%)

**Figure 1:**
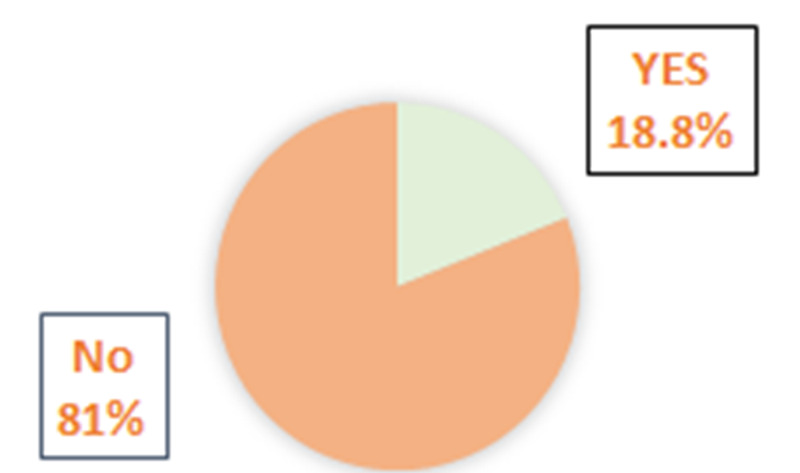
Cancer family history

When evaluated on the type of cancer (Table 2), 10.4% suffered thyroid cancer, 45.5% suffered breast cancer, 6.5% suffered lung cancer, 6.5% suffered colon cancer, 14.3% suffered blood cancer, 7.1% suffered bone cancer, and 5.2% suffered ovarian cancer. With regards to the treatment types used by the participants, 62.3% reported undergoing surgery, 79.2% used chemotherapy, 55.2% used radiation, 29.9% reported hormonal treatment, and 13% reported immunotherapy. On when symptoms occur, 27.9% said after diagnosis, 75.3% said within the treatment, and 37% said after treatment. Over 18.8% had a family history and 93.5% had supportive family and friends (Figure 1).

**Table 2:** Demographic data description

Health situation		N%
Cancer types		
Thyroid cancer	Suffered	16 (10.4%)
	Did not suffer	138 (89.6%)
Breast cancer	Suffered	70 (45.5%)
	Did not suffer	84 (54.5%)
Lung cancer	Suffered	10 (6.5%)
	Did not suffer	144 (93.5%)
Colon cancer	Suffered	10 (6.5%)
	Did not suffer	144 (93.5%)
Blood cancer	Suffered	22 (14.3%)
	Did not suffer	132 (85.7%)
Bone cancer	Suffered	11 (7.1%)
	Did not suffer	143 (92.9%)
Ovarian cancer	Suffered	8 (5.2%)
	Did not suffer	146 (94.8%)
Treatment types		
Surgery	Yes	96 (62.3%)
	No	58 (37.7%)
Chemotherapy	Yes	122 (79.2%)
	No	32 (20.8%)
Radiation	Yes	85 (55.2%)
	No	69 (44.8%)
Hormonal	Yes	46 (29.9%)
	No	108 (70.1%)
Immunotherapy	Yes	20 (13.0%)
	No	134 (87.0%)
When symptoms occur		
Before diagnosis	Yes	43 (27.9%)
	No	111 (72.1%)
Within treatment	Yes	116 (75.3%)
	No	38 (24.7%)
After treatment	Yes	57 (37.0%)
	No	97 (63.0%)
Family history	Yes	29 (18.8%)
	No	125 (81.2%)
Family and friends support	They were supportive	144 (93.5%)
	They were not	10 (6.5%)

According to Table 3, there was a significant correlation between cancer survivors who did not suffer irritability and their education level as most of them were educated at a university (P=0.014 <0.05). Furthermore, there was a significant relationship between cancer survivors who did not suffer from irritability and their socioeconomic status as most of them owned houses (P=0.005 <0.05). On the other hand, there was no significant correlation between the above-mentioned cancer symptoms and the survivor’s income level, job, age, and gender (all P>0.05).

**Table 3:** Socioeconomic status cross symptoms

Socioeconomic status cross symptoms	Irritable (P-value)	Lack of confidence (P-value)	Sadness (P-value)
Gender	0.659	0.512	0.308
Age	0.625	0.719	0.369
Education level	0.014	0.641	0.695
Accommodation type	0.005	0.130	0.227
Job	0.192	0.995	0.895
Income	0.068	0.764	0.235

According to Table 4, there was a significant correlation between cancer survivors who did not suffer from frustration and their socioeconomic status. Most survivors who did not suffer from frustration show that their income level was high (P=0.008 <0.05). In addition, the study failed to establish a significant relationship between the above-mentioned cancer symptoms and survivor’s, job, age, accommodation type, and gender (all P>0.05).

**Table 4:** Socioeconomic status cross symptoms

Socioeconomic status cross symptoms	Negative thought (P-value)	Avoid thinking about your experience (P-value)	Having anger outbursts (P-value)
Gender	0.187	0.953	0.525
Age	0.703	0.390	0.389
Education level	0.190	0.193	0.809
Accommodation type	0.270	0.543	0.068
Job	0.926	0.122	0.349
Income	0.008	0.485	0.127

According to Table 5, there was a significant correlation between cancer survivors who did not suffer from hopelessness about the future and their age as easily gleaned from the table. Most survivors who did not suffer from hopelessness about the future were between 40 and 50 years old (P=0.0001 <0.05). The study established a significant relationship between cancer survivors who did not suffer from loss of interest in enjoyable activities and their socioeconomic status as most of them owned their own houses (P=0.039 <0.05). In addition, there was a significant relationship between cancer survivors who did not suffer from hopelessness about the future and their socioeconomic status as most of them owned their own houses (P=0.017 <0.05).

**Table 5:** Socioeconomic status cross symptoms

Socioeconomic status cross symptoms	Negative thought (P-value)	Loss of interest in enjoyable things (P-value)	Hopelessness about the future (P-value)
Gender	0.780	0.599	0.554
Age	0.354	0.107	0.0001
Education level	0.447	0.938	0.799
Accommodation type	0.960	0.039	0.017
Job	0.443	0.145	0.562
Income	0.130	0.673	0.601

According to Table 6, there was a significant relationship between cancer survivors who did not suffer from feeling detached from family and friends and their socioeconomic status as most of them were owned houses level (P=0.002 <0.05).

**Table 6:** Socioeconomic status cross symptoms

Socioeconomic status cross symptoms	Feeling detached from family and friends (P-value)
Gender	0.709
Age	0.280
Education level	0.269
Accommodation type	0.022
Job	0.935
Income	0.953

According to Table 7, there was a significant correlation between cancer survivors who did not suffer from obsessive-compulsive and their socioeconomic status as most of them owned their own houses (P=0.022 <0.05). In addition, there was a significant correlation between cancer survivors who did not think about suicide and their job status (P=0.016 <0.05).

**Table 7:** Socioeconomic status cross symptoms

Socioeconomic status cross negative thoughts	Suicide (P-value)	Obsessive-compulsive disorder (P-value)	Sadness (P-value)
Gender	0.343	0.569	0.533
Age	0.008	0.347	0.252
Education level	0.483	0.734	0.499
Accommodation type	0.399	0.022	0.624
Job	0.016	0.857	0.630
Income	0.713	0.179	0.744

According to Table 8, there was a clear significant correlation between cancer survivors and the above-mentioned negative thoughts with their family and friends' support. We can easily notice that most of the survivors who did not think about suicide and obsessive-compulsive disorder had supportive family and friends for them (P<0.05). On the other hand, for most survivors who had a fear of recurrence, their families and friends did not support them (P=0.012 <0.05). There was a significant correlation between cancer survivors who treated with chemotherapy and avoid thinking talking about their experience, 88 (72.1%) of them did not avoid thinking talking about their experience. In addition, there was a significant correlation between cancer survivors who were treated by immunotherapy and frustration, avoid thinking talking about their experience.

**Table 8:** Treatment types and family support cross negative thoughts

Treatment types and family support cross-negative thoughts	Suicide (P-value)	Obsessive-compulsive disorder (P-value)	Fear of recurrence (P-value)	Nothing (P-value)
Surgery	0.230	0.772	0.396	0.632
Chemotherapy	0.321	0.665	0.616	0.654
Radiation	0.964	0.916	0.918	0.383
Hormonal	0.379	0.442	0.568	0.720
Immunotherapy	0.595	0.295	0.374	0.736
Family Support	0.004	0.0001	0.012	0.870

According to Table 9, there was a significant correlation between breast cancer survivors and being fear of recurrence (P=0.023 <0.05). In addition, there is a significant correlation between blood cancer survivors and thinking about suicide (P=0.030 <0.05).

**Table 9:** Cancer types cross negative thoughts

Cancer types cross negative thoughts	Suicide (P-value)	Obsessive-compulsive disorder (P-value)	Fear of recurrence (P-value)
Thyroid cancer	0.884	0.356	0.147
Breast cancer	0.209	0.888	0.023
Lung cancer	0.717	0.475	0.012
Colon cancer	0.364	0.475	0.070
Blood cancer	0.030	1.000	0.211
Bone cancer	0.795	0.453	0.395
Ovarian cancer	0.546	0.526	0.819

There was a significant correlation between patients who did not suffer from colon cancer and (irritability, lacking confidence, and sadness), 77 (53.3%) of them suffered from irritability, 102 (70.8%) did not suffer from lacking confidence, and 86 (59.7%) suffered from sadness (P<0.05). In addition, there was a significant relationship between patients who did not suffer from ovarian cancer and did not suffer from lacking confidence (Table 10).

**Table 10:** Cancer types, family support, and fear of recurrence cross negative thoughts

Cancer types, family support, and fear of reoccurrence negative thoughts	Suicide (P-value)	Lack of confidence (P-value)	Sadness (P-value)
Surgery	0.678	0.293	0.053
Chemotherapy	0.337	0.224	0.666
Radiation	0.898	0.247	0.517
Hormonal	0.887	0.829	0.726
Immunotherapy	0.723	0.434	0.735
Family support	0.932	0.095	0.668
Thyroid cancer	0.676	0.161	0.296
Breast cancer	0.346	0.290	0.423
Lung cancer	0.221	0.593	0.277
Colon cancer	0.041	0.045	0.002
Blood cancer	0.895	1.000	0.259
Bone cancer	0.396	0.482	0.892
Ovarian cancer	0.423	0.022	0.278
Fear of recurrence	0.818	0.223	0.065

The study also notices that most of the survivors who did not avoid thinking talking about their experience, and did not have angry outbursts, their family and friends were supportive to them (P<0.05; Table 11).

**Table 11:** Cancer and treatment, family support, fear of recurrence cross negative thoughts

Cancer and treatment types, family support, fear of recurrence cross negative thoughts	Frustrated (P-value)	Avoid thinking about your experience (P-value)	Having anger outbursts (P-value)
Surgery	0.906	0.217	0.395
Chemotherapy	0.746	0.040	0.634
Radiation	0.540	0.289	0.394
Hormonal	0.058	0.891	0.708
Immunotherapy	0.044	0.025	0.842
Family support	0.087	0.007	0.025
Thyroid cancer	0.829	0.279	0.555
Breast cancer	0.229	0.801	0.330
Lung cancer	0.306	0.126	0.379
Colon cancer	0.729	0.566	0.067
Blood cancer	0.730	0.323	0.946
Bone cancer	0.925	0.737	0.0001
Ovarian cancer	0.095	0.671	0.470
Fear of recurrence	0.003	0.096	0.931

According to Table 12, we can easily notice that most of the survivors who were not hopeless about the future had supportive family and friends (P<0.05). In addition, there was a significant correlation between fear of recurrence and suffering from negative thoughts, 50 (49%) were suffering from fear of recurrence and negative thoughts at the same time.

**Table 12:** Cancer and treatment types, family support, and fear of recurrence cross negative thoughts

Cancer and treatment types, family support, and fear of recross negative thoughts	Negative thoughts (P-value)	Loss of interest in enjoyable things (P-value)	Hopelessness about the future (P-value)
Surgery	0.559	0.616	0.862
Chemotherapy	0.650	0.741	0.244
Radiation	0.559	0.818	0.739
Hormonal	0.515	0.041	0.754
Immunotherapy	0.929	0.818	0.014
Family support	0.204	0.409	0.040
Thyroid cancer	0.435	0.201	0.432
Breast cancer	0.268	0.001	0.531
Lung cancer	0.545	0.409	0.609
Colon cancer	0.468	0.628	0.071
Blood cancer	0.640	0.035	0.938
Bone cancer	0.75	0.263	0.673
Ovarian cancer	0.591	0.880	0.911
Fear of recurrence	0.004	0.366	0.642

From Table 13, the study established that for most of the survivors who were treated by chemotherapy their family and friends were supportive to them (P<0.05).

**Table 13:** Cancer and treatment types, family support, fear of recurrence cross negative thoughts

Cancer and treatment types, family support, fear of recurrence cross negative thoughts	Feeling detached from family and friends (P-value)
Surgery	0.065
Chemotherapy	0.545
Radiation	0.486
Hormonal	0.882
Immunotherapy	0.484
Family support	0.066
Thyroid cancer	0.895
Breast cancer	0.858
Lung cancer	0.066
Colon cancer	0.033
Blood cancer	0.287
Bone cancer	0.006
Ovarian cancer	0.054
Fear of recurrence	0.744

## Discussion

The findings of this study are based on a study conducted among 154 cancer survivors at the King Fahad and King Abdul-Aziz hospitals in Jeddah. Accumulating data over the past decade indicate a significant relationship between socioeconomic status and depression in cancer survivors; they found a high association between low socioeconomic status and depression which may be due to less access to medical resources [10]. Our study finds that there is no association between cancer symptoms (irritability, lack of confidence, and sadness) and survivor’s level of income. However, frustration has a significant relationship with the survivor’s level of income. This finding agrees with a study published in 2019, done by Alleaume et al. in a representative sample of 4,174 cancer survivors, which aims to find the cost impact on cancer survivors in France; the study found out that inequality in economic well-being persists long after cancer treatment [22].This could explain the frustration experienced by participants in this study. The study further established a significant relationship between the type of accommodation and the loss of interest in enjoyable activities. This was also the case with the patient being hopeless about the future. The study also established a significant relationship between survivors who had a feeling of detachment from family and friends and their social-economic status measured with the type of accommodation. A significant relationship was also established between social-economic status (the type of house) and the survivors experiencing the obsessive-compulsive disorder. These findings agree with a study published in 2016 done by DiMartino et al. with a sample size reach to 1320 cancer survivors. The study was conducted on the social-economic status and follow-up care for cancer survivors where the researchers established a significant association between socioeconomic status and the probability of a cancer survivor reporting follow up discussions with a provider. The study went ahead to recommend the establishment of survivorship care plans for the socioeconomically disadvantaged cancer survivors [23]. Bevilacqua et al. did a study of over 1012 survivors of different types of cancer who referred to Survivorship Clinics at Memorial Sloan Kettering Cancer Center to find out if there is a relation between age and gender to depression among cancer survivors. They found that there are no differences in depression between older (65 and above years old) and younger adult (30-55 years old) cancer survivors but there is a small higher score for the younger to develop depression, while in the case of gender; women reported greater rates of depression score than men [3]. While Another study was done in 2015 by Champion et al. compared younger (diagnosed at age 45 years or before) and older breast cancer survivors (diagnosed between 55 and 70 years) and age-matched controls on the specific and overall quality of life domains. The study revealed that Younger survivors reported more depression than age-matched control (p<0.001) and reported more depressive symptoms (p<0.001) than older survivors [4]. Our study did not establish a significant relationship between gender and symptoms. A similar finding was also replicated for age and occupation where these two factors were not seen to have a significant association with the symptoms. These findings agree with a study published in 2015 done by Din et al., in investigating gender and age variation in cancer patients which failed to find significant variations that could be attributed to age and gender [24]. A different study published in 2010 done by Shariat et al. on age and gender concluded that gender and age were particular to the type of cancer and could not be generalized [25]. The study established a significant association between the patient’s job status and not thinking about suicide. This finding agrees with a study published in 2019 and done by Zaorsky et al. with a total of 8.6 million cancer patients were included. The study found out that suicide or thoughts about suicide were susceptible to many factors such as the function of the affected site, age, gender, marital status, and time after diagnosis. As a result, this study tends to follow that job status is more likely to be a factor when it comes to thoughts about suicide [26]. Family support has been seen to play a significant role in preventing most of the symptoms among cancer survivors. The study has established a significant relationship between family support and the patients experiencing thoughts of suicide, obsessive-compulsive disorder, and fear of recurrence. These findings agree with a study published in 2011 and done by Muhamad et al.; the study was conducted among 400 breast cancer survivors in Malaysia where it was found out that collaborative decision making would help in the recovery process [27]. The study found out that patients who underwent chemotherapy were less likely to talk about their experiences. Further, those who underwent immunotherapy had a significant relationship with being frustrated and were also not willing to talk about their experiences. There was also a relationship between a patient having anger outbursts and not willing to talk about their experiences. Although not many studies are available on this subject, the articles published generally agree with the findings. The study found a significant relationship between most survivors and fear recurrence, more so with breast cancer survivors. These findings agree with a study published in 2019 and done by Sun et al. conducted among 249 young adult cancer patients on fear of occurrence where fear of recurrence, anxiety, and depressive symptoms were the most reported problems among young adult cancer patients [28]. This study was not without its limitations. First, the sample size was a bit small given the difficulty and uniqueness of the sample. This largely hinders the generalization of the results to a larger population. Second, the gender composition was highly biased towards female survivors and this could bias the generalization and may not be representative of both genders.

## Conclusions

The study concludes that post-cancer treatment symptoms are highly affected by other factors such as socioeconomic status as well as family support. A clear significance may suggest a correlation between survivors' socioeconomic status such as university education level, owned house, high-income level, and their impact as most of them did not suffer from negative thoughts. Also, we can easily notice the importance of family and social support as a risk factor. Most of the survivors who were not hopeless about the future also did not avoid thinking talking about their experience, who did not have angry outbursts, and who did not think about suicide and obsessive-compulsive disorder, they all had support from their family and friends. On the other hand, for most survivors who had a fear of recurrence, their families and friends did not support them. As a result, the study recommends the establishment of educational materials on post-treatment symptoms, care-plans, and support groups for cancer survivors.

## References

[1] Massie MJ (2004). Prevalence of depression in patients with cancer. J Natl Cancer Inst Monogr.

[2] (2020). What is depression?. https://www.psychiatry.org/patients-families/depression/what-is-depression.

[3] Bevilacqua LA, Dulak D, Schofield E (2018). Prevalence and predictors of depression, pain, and fatigue in older- versus younger-adult cancer survivors. Psychooncology.

[4] Champion VL, Wagner LI, Monahan PO (2014). Comparison of younger and older breast cancer survivors and age-matched controls on specific and overall quality of life domains. Cancer.

[5] Miaskowski C (2004). Gender differences in pain, fatigue, and depression in patients with cancer. J Natl Cancer Inst Monogr.

[6] Winther D, Nygaard TK, Horsbøl TA (2017). Associations between education and physical functioning and pain in adult Danish cancer survivors. Acta Oncol.

[7] Üstündağ S, Zencirci AD (2015). Factors affecting the quality of life of cancer patients undergoing chemotherapy: a questionnaire study. Asia Pac J Oncol Nurs.

[8] Can G, Erol O, Aydiner A, Topuz E (2009). Quality of life and complementary and alternative medicine use among cancer patients in Turkey. Eur J Oncol Nurs.

[9] Taskila T, Lindbohm ML (2007). Factors affecting cancer survivors&apos; employment and work ability. Acta Oncol.

[10] Fagundes C, Jones D, Vichaya E, Lu C, Cleeland CS (2014). Socioeconomic status is associated with depressive severity among patients with advanced non-small-cell lung cancer: treatment setting and minority status do not make a difference. J Thorac Oncol.

[11] Hu T, Xiao J, Peng J, Kuang X, He B (2018). Relationship between resilience, social support as well as anxiety/depression of lung cancer patients: a cross-sectional observation study. J Cancer Res Ther.

[12] Litzelman K, Yabroff KR (2015). How are spousal depressed mood, distress, and quality of life associated with risk of depressed mood in cancer survivors? Longitudinal findings from a national sample. Cancer Epidemiol Biomarkers Prev.

[13] Jacob A, Veeraiah S (2017). Fear of recurrence among cancer survivors. J Cancer Res Ther.

[14] (2020). Saudi Arabia cancer country profile. https://www.who.int/cancer/country-profiles/sau_en.pdf.

[15] Kim MS, Kim SY, Kim JH, Park B, Choi HG (2017). Depression in breast cancer patients who have undergone mastectomy: a national cohort study. PLoS One.

[16] Reich M, Lesur A, Perdrizet-Chevallier C (2008). Depression, quality of life and breast cancer: a review of the literature. Breast Cancer Res Treat.

[17] Averyt JC, Nishimoto PW (2014). Psychosocial issues in colorectal cancer survivorship: the top ten questions patients may not be asking. J Gastrointest Oncol.

[18] Mols F, Schoormans D, de Hingh I, Oerlemans S, Husson O (2018). Symptoms of anxiety and depression among colorectal cancer survivors from the population‐based, longitudinal PROFILES Registry: Prevalence, predictors, and impact on quality of life. Cancer.

[19] Ginsburg A (2008). Cancer-related depression and potential pharmacologic therapies. Proc (Bayl Univ Med Cent).

[20] Smith HR (2015). Depression in cancer patients: pathogenesis, implications and treatment (review). Oncol Lett.

[21] Rowland JH, Desmond KA, Meyerowitz BE, Belin TR, Wyatt GE, Ganz PA (2000). Role of breast reconstructive surgery in physical and emotional outcomes among breast cancer survivors. J Natl Cancer Inst.

[22] Alleaume C, Bendiane MK, Peretti-Watel P, Bouhnik AD (2019). Inequality in income change among cancer survivors five years after diagnosis: evidence from a French national survey. PLoS One.

[23] DiMartino LD, Birken SA, Mayer DK (2017). The relationship between cancer survivors’ socioeconomic status and reports of follow-up care discussions with providers. J Cancer Educ.

[24] Din NU, Ukoumunne OC, Rubin G, Hamilton W, Carter B, Stapley S, Neal RD (2015). Age and gender variations in cancer diagnostic intervals in 15 cancers: analysis of data from the UK clinical practice research datalink. PLoS One.

[25] Shariat SF, Sfakianos JP, Droller MJ, Karakiewicz PI, Meryn S, Bochner BH (2010). The effect of age and gender on bladder cancer: a critical review of the literature. BJU Int.

[26] Zaorsky NG, Zhang Y, Tuanquin L, Bluethmann SM, Park HS, Chinchilli VM (2019). Suicide among cancer patients. Nat Commun.

[27] Muhamad M, Afshari M, Kazilan F (2011). Family support in cancer survivorship. Asian Pac J Cancer Prev.

[28] Sun H, Yang Y, Zhang J, Liu T, Wang H, Garg S, Zhang B (2019). Fear of cancerrecurrence, anxiety and depressive symptoms in adolescent and young adultcancer patients. Neuropsychiatr Dis Treat.

